# Necroptosis in Hepatosteatotic Ischaemia-Reperfusion Injury

**DOI:** 10.3390/ijms21165931

**Published:** 2020-08-18

**Authors:** Raji Baidya, Darrell H. G. Crawford, Jérémie Gautheron, Haolu Wang, Kim R. Bridle

**Affiliations:** 1 Faculty of Medicine, The University of Queensland, Brisbane, Queensland QLD 4006, Australia; r.baidya@uqconnect.edu.au (R.B.); d.crawford@uq.edu.au (D.H.G.C.); 2 Gallipoli Medical Research Institute, Brisbane, Queensland QLD 4120, Australia; h.wang21@uq.edu.au; 3 Sorbonne University, Inserm, Centre de Recherche Saint-Antoine (CRSA), 75012 Paris, France; jeremie.gautheron@inserm.fr; 4 Institute of Cardiometabolism and Nutrition (ICAN), 75013 Paris, France; 5 Diamantina Institute, The University of Queensland, Brisbane, Queensland QLD 4102, Australia

**Keywords:** ischaemia-reperfusion injury, necroptosis, liver transplantation, steatosis, non-alcoholic fatty liver disease

## Abstract

While liver transplantation remains the sole treatment option for patients with end-stage liver disease, there are numerous limitations to liver transplantation including the scarcity of donor livers and a rise in livers that are unsuitable to transplant such as those with excess steatosis. Fatty livers are susceptible to ischaemia-reperfusion (IR) injury during transplantation and IR injury results in primary graft non-function, graft failure and mortality. Recent studies have described new cell death pathways which differ from the traditional apoptotic pathway. Necroptosis, a regulated form of cell death, has been associated with hepatic IR injury. Receptor-interacting protein kinase 3 (RIPK3) and mixed-lineage kinase domain-like pseudokinase (MLKL) are thought to be instrumental in the execution of necroptosis. The study of hepatic necroptosis and potential therapeutic approaches to attenuate IR injury will be a key factor in improving our knowledge regarding liver transplantation with fatty donor livers. In this review, we focus on the effect of hepatic steatosis during liver transplantation as well as molecular mechanisms of necroptosis and its involvement during liver IR injury. We also discuss the immune responses triggered during necroptosis and examine the utility of necroptosis inhibitors as potential therapeutic approaches to alleviate IR injury.

## 1. Introduction

Liver transplantation still remains the only curative option for the patients with end-stage liver disease. Over the past few decades, advances in surgical techniques, development of immunosuppressive agents, proper donor management and improved organ preservation methods along with better post-operative care have improved the transplantation survival rate and quality of life of patients. However, the increasing incidence of chronic liver diseases and the emergence of non-alcoholic fatty liver disease (NAFLD) has increased the number of patients requiring liver transplantation, increased the number of patients on the waiting list and, hence, increased waiting times for recipients [1,2,3].

Due to the limited number of donor livers and increasing demand for liver transplantation, the expansion of the donor pool of livers has become an urgent priority and the use of extended criteria donors (ECDs) liver is essential [2,4,5]. Marginal organs need to be accurately evaluated before being discarded as many remain underused [6]. Mainly, ECD livers can be classified into two groups [4]. Firstly, a liver that poses a risk of transmitting diseases, e.g., hepatitis B, hepatitis C, active bacterial infection or malignancy to the recipient [4,5]. The second group are those whereby the liver poses a risk of graft non-function and/or high technical difficulties during transplantation such as livers from steatotic donors, elderly donors, non-heart-beating donors, split grafts and donation after cardiac death (DCD) [4]. Hence, marginal or extended criteria donor livers are defined as an organ with an increased risk of primary non-function (PNF) or delayed graft failure that causes an increased risk of morbidity or mortality in the recipient [4,7]. Though steatotic livers are potential candidates for transplantation, they are known to be remarkably vulnerable to ischaemia-reperfusion (IR) injury [8,9]. Since IR starts a series of cellular responses in the traumatized cells, this could result in irreversible injury and cell death. Recently, necroptosis, a novel form of cell death, has been extensively linked with IR injury. Traditionally, necroptosis is considered to be an accidental and uncontrolled event. However, a number of recent studies have described its involvement during IR injury.

ECD livers have been previously associated with necroptosis. For instance, necroptosis has been recently directly linked with liver IR injury in an aged mouse model study [10]. This study revealed that IR injury caused excessive endoplasmic reticulum stress resulting in hepatocyte necroptosis [10]. Similarly, prolonged cold ischaemia also triggers necroptotic cell death during IR injury [11]. The use of controlled DCD liver has been promoted as a major strategy to improve the number of liver donors. However, the use of DCD livers has been associated with the development of non-anastomotic biliary strictures as DCD livers are more susceptible to IR injury and increased graft dysfunction [12,13]. During retrieval of a DCD liver the organ enters a phase of ischaemia which can trigger the release of stress factors resulting in secretion of endotoxin and gut bacteria into the portal circulation [14]. These endotoxins potentially translocate to the liver during cold perfusion [15]. The aggregation of these endotoxins can result in graft failure in animal models [16]. However, whether necroptosis is involved in this injury has not been fully examined.

In this review, we focus on necroptosis pathways during IR injury in the steatotic liver. We summarise the impact of hepatic steatosis during liver transplantation as well as molecular mechanisms of necroptosis and its involvement during liver IR injury. We also discuss the immune responses triggered during necroptosis and examine the use of inhibitors of necroptosis as potential therapeutic approaches to alleviate IR injury.

## 2. Materials and Methods

We used NCBI PubMed and ScienceDirect databases for literature research. Articles were searched using the terms: necroptosis and liver ischemia/reperfusion injury, hepatic ischemia/reperfusion injury, liver steatosis, liver transplantation, damage-associated molecular pattern molecules (DAMPs) signals, pharmacological/therapeutic approaches and organ preservation. Only publications in English were included. Articles were screened to identify potentially relevant studies. Reference lists of retrieved literature were also searched for relevant publications.

## 3. Effect of Hepatic Steatosis during Liver Transplantation

With the increasing global rate of obesity, 10–24% of the world population is considered to have hepatic steatosis, and it is predicted to continuously rise in the future [17]. Hepatic steatosis is frequent in both deceased and live organ donors, and multiple studies have reported an estimated prevalence of steatosis in liver procurements of 9–26% [18]. Further, 5–10% of donated livers are discarded due to the fact of steatosis [19]. This may represent a large pool of donor livers and, hence, the use of fatty livers for graft transplantation has become a topic of great interest.

Histologically, hepatic steatosis has been divided quantitatively and qualitatively [20]. Quantitatively, steatotic livers have been graded based on fat infiltration of hepatocytes as mild (<30%), moderate (30–60%) and severe >60% [21]. Whereas the qualitative classification is based on the size of intra-cytoplasmic fat deposited in the hepatocytes [20]. A majority of studies have shown that the steatotic liver displays a range of complications after transplantation. In general, mildly steatotic (<30%) livers have been used by transplant centres across the globe with high success rates [22]. On the other hand, moderately steatotic livers (30–60%) pose a 0–75% rate of PNF, hence caution is generally adopted while selecting such livers, and they are often discarded if additional risks are present [23]. Qualitatively, liver steatosis is classified into microvesicular and macrovesicular steatosis [20,23]. Microvesicular steatosis is described as the accumulation of tiny lipid vesicles in the cytoplasm without dislocating the nucleus. Macrovesicular steatosis consists of a single large fat vacuole in the cytoplasm displacing the nucleus to the periphery [20].

Both micro- and macrosteatosis are often simultaneously present in hepatocytes in various proportions. Generally, the use of livers with microsteatosis has no effect on transplant outcomes [24,25]. However, recent studies have shown that severe microsteatosis has been linked to delayed liver function [26]. Use of livers with mild macrosteatosis (<30%) is considered to be safe for transplantation, as many studies have shown similar functional results to that of non-steatotic liver [21,22]. In contrast, severely macrosteatotic livers (>60%) have been deemed as unsuitable for transplantation as they pose a high risk of PNF and increased recipient mortality [21,27,28]. Currently, there is debate whether moderate macrosteatosis livers (30–60%) are acceptable for transplantation. Some studies have demonstrated similar PNF rates and survival compared to non-steatotic livers [21,28,29,30], while others have shown an increased rate of complications, PNF and death [28,31]. However, the use of livers with moderate macrosteatosis is probably justified given the increasing number of patient deaths on liver transplantation waiting lists.

## 4. Microvascular Dysfunction During IR Injury of Steatotic Livers and Clinical Consequences

During transplantation, donor livers are destined to be exposed to IR injury at various stages including after organ retrieval, then during cold storage and finally during implantation to the recipient. ECD livers, specifically steatotic livers, are remarkably vulnerable to IR injury. Fat globules present in the hepatocytes are known to exaggerate microcirculatory disturbance in the liver [32,33]. Large fat droplets in steatotic hepatocytes are responsible for compressing the sinusoidal architecture and, consequently, obstructing intrahepatic blood flow [32], and parenchymal perfusion is greatly reduced. The random rupturing of hepatocytes during transplantation can cause the release of these large fat droplets into the hepatic microcirculation causing clogging, distortion and disruption of the sinusoidal space increasing congestion, focal haemorrhage as well as hepatocyte necrosis [34,35]. Hence, acute and chronic hepatic complications including PNF, delayed graft dysfunction, postsurgical biliary complications and increased morbidity are often evident [21,27,36,37]. Further, studies have also shown that the abnormally high ratio of omega-6:omega-3 polyunsaturated fatty acid in macrosteatotic liver can contribute to microvascular dysfunction and hepatic injury during IR [38]. This vulnerability to microcirculatory impairment during IR is linked to lower omega-3 content [38] as well as impaired sinusoidal perfusion combined with oxidative stress-associated hepatocyte damage and Kupffer cell activation [39]. Interestingly, dietary supplementation of omega-3 polyunsaturated fatty acid can reduce the IR injury in steatotic liver by attenuating Kupffer cell activity [38].

## 5. Mechanisms of IR Injury during Liver Transplantation

### 5.1. Ischaemic Phase

**Figure 1:**
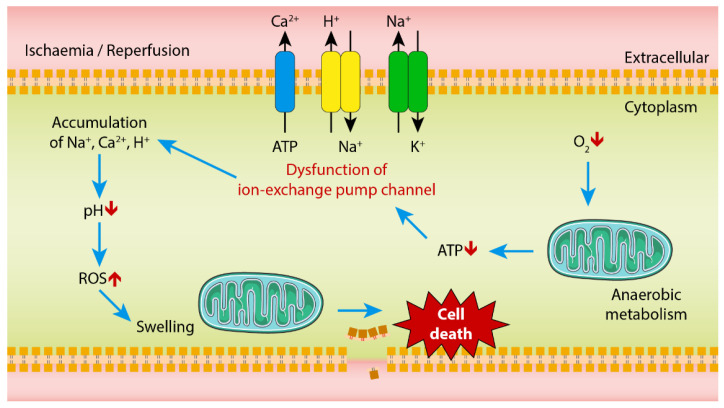
Overview of the process of ischemia-reperfusion (IR) injury. Upon depletion of oxygen during the ischaemic stage, mitochondria initiate anaerobic metabolism and ATP production decreases. Further, ion-exchange pump channel dysfunction and pH level decreases leading to cell swelling. During the reperfusion stage, mitochondrial swelling and accumulation of H^+^, Na^+^ and K^+^ result in oxidative stress leading to the excessive production of ROS. This induces cell injury, leading to cell death. The figure is modified from Reference [48].

IR injury contributes to 10% of early graft failures [40] and is also responsible for non-anastomotic biliary strictures following graft transplant [41,42,43,44] and is the underpinning common event in liver transplantation. At the cellular level, the pathological features of ischaemia results from several insults. Hypoxia is caused by obstruction of blood flow and subsequent loss of oxygen causes the disruption of the electron transport chain in mitochondria [45] (Figure 1). The loss of oxygen triggers the anaerobic metabolism where adenosine triphosphate (ATP) breaks down into adenosine diphosphate (ADP) and adenosine monophosphate (AMP) which could result in the lactic acid accumulation and decreased pH level in mitochondria [46]. Moreover, there is a reduction in nutrient supply and an increase in waste accumulation due to the impairment in blood circulation [47]. As ATP depletes, the ATP-dependent sodium potassium (Na^+^-K^+^) pump channel is altered during the ischaemic phase and as a result, Na^+^ ions accumulate intracellularly [48]. Excessive Na^+^ inside the cell reduces the sodium–hydrogen exchange channel. In the endoplasmic reticulum (ER), the calcium pump (Ca^2+^-ATPase pump) channel dysfunction restricts Ca^2+^ intake. Hyper-osmolarity is caused by the accumulation of hydrogen (H^+^), Na^+^ and Ca^2+^ ions inside the cell leading to oedema and swelling of hepatocytes, Kupffer cells and sinusoidal endothelial cells [49]. Mitochondrial swelling, increase in cellular volume and bleb formation at the plasma membrane are considered the initial hepatocellular alterations during early stages of ischaemia [50].

### 5.2. Reperfusion Phase

Reperfusion following restoration of blood supply further elevates the cellular injury [48] (Figure 1). Mitochondria play an essential role in IR due to the fact of their central role in ATP production and reactive oxygen species (ROS) formation. During the early phase of reperfusion, the leakage of extensive electrons from disrupted electron transport chains results in the burst of ROS from mitochondria and initiates further damage downstream. Further Kupffer cell activation at this stage increases their release of ROS and proinflammatory cytokines, including tumour necrosis factor alpha (TNFα), interlukin-1 (IL-1), interferon-γ (IFN-γ) and interlukin-12 (IL-12) [51,52]. Normally, in mitochondria, when a small amount of oxygen is reduced to ROS, it is neutralized by the antioxidant superoxide dismutase (MnSOD) into hydrogen peroxide and later into water by glutathione peroxide (GPx) [53]. However, during ischaemia, as more ROS are produced, the capability of MnSOD and GPx to neutralize ROS is reduced resulting in excessive oxidative stress causing endothelial dysfunction and DNA damage. Hence more toxic hydroxy radical (OH^.^) is formed and, ultimately, unavoidable injury takes place when the ionic gradient across the plasma membrane disrupts the ionic balance and the plasma membrane bursts. This releases all intracellular organelles into the surrounding area resulting in the necrotic form of cell death and release of local damage inflammatory responses [54].

## 6. Necroptosis

Apoptosis was known as the key cell death pathway playing a critical role in tissue development and morphogenesis of multi-cellular eukaryotes [55] until other forms of regulated cell death pathways were identified. At the cellular level, the hallmark features of apoptosis include chromatin condensation, nuclear fragmentation, DNA damage, cytoplasmic shrinkage and the formation of apoptotic bodies [56,57]. In contrast, necrosis is generally considered to be an uncontrolled event which is characterised by the sudden loss of membrane integrity resulting in the spill of extracellular content triggering inflammation and DAMPs signals [58,59]. Unlike apoptosis, a striking feature of necrosis is organelle swelling, as the cell fails to maintain homoeostasis within its environment [58,60]. In the mid-19th century, surgical pathologists initially used the term “necrosis” while describing destructive tissue incidents [61,62]. Since then, there has been uncertainty surrounding whether necrosis is a gene-driven programmed process or it is simply an accidental and passive event caused by environmental circumstances.

Necroptosis is a form of regulated necrosis and defined as a novel form of programmed cell death but is biochemically distinct from apoptosis [63,64,65]. At the cellular level, like in the case of necrosis, necroptosis involves cytoplasmic granule formation followed by organelle swelling that leads to loss of membrane integrity and finally rupture of the plasma membrane [66,67,68]. Unlike apoptotic cells where macrophages are involved for phagocytosis, during necrotic cell death there is release of intracellular organelles and DAMPs [69,70]. Hence, inflammatory signals are disseminated in adjacent cells. Necroptosis participates in several pathological disorders that include viral infection [71], pancreatitis [72], bowel diseases [73] and IR injury [8,74,75].

### 6.1. Molecular Mechanism of Necroptosis

In recent years, many proteins and processes have been coined as instigator, executor and effectors of necroptosis. Only recently, a better understanding of the key molecular mechanisms of necroptosis have emerged. 

#### 6.1.1. Initiation of Necroptosis by the Death Receptor

Necroptosis can be activated by a number of different receptors. The necroptotic form of cell death can potentially be activated by either extracellular or intracellular signals. Death receptors, such as tumour necrosis factor receptor superfamily member 1 (TNFR1), fas cell surface death receptor (FAS), Z-DNA binding protein 1 (ZBP1) or pathogen recognition receptor (PRR), can recognize such disruption in the form of death signals [65,75,76,77], toll-like receptor (TLR) 3 or TLR4 [78,79] and interferons [76].

#### 6.1.2. TNFR1

**Figure 2:**
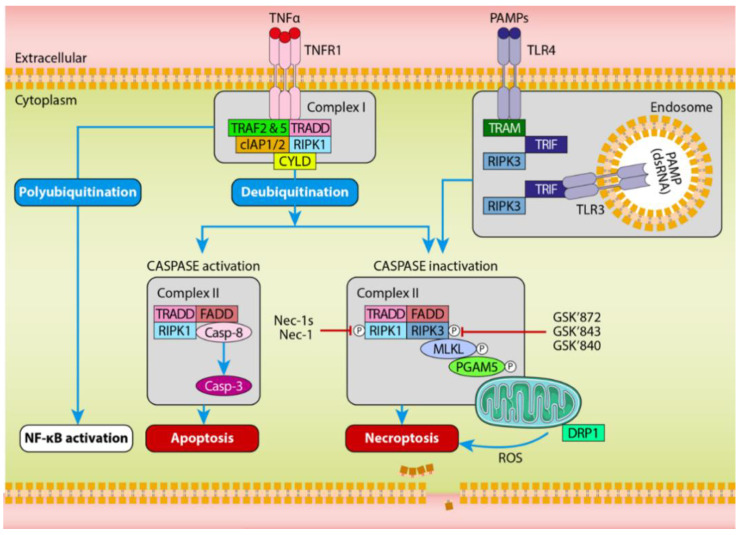
TNFα-induced cell death pathway. TNFα stimulates TNFR1 to generate complex I by recruiting TRADD, TRAF2 and 5, RIPK1 and cIAP1/2. Polyubiquitination of RIPK1 in complex I will activate the NF-κB pathway, whereas polyubiquitination of RIPK1 by CLYD shifts complex I to cytoplasm to form complex II. Activation of CASPASE8 will result in activation of CASPASE3 and cells undergo apoptosis. Upon inhibition of CASPASE8, activation and phosphorylation of RIPK1 leads to recruitment of RIPK3 and further recruits MLKL to form the necrosome. Further activation of PGAM5 and DRP1 results in ROS production in mitochondria and induces necroptosis. Activation of TLR3/ TLR4 by PAMPs or LPS, activates Toll–IL-1 receptor domain-containing adaptor-inducing IFN-β (TRIF) and RIPK3 binding and triggers necroptosis. Abbreviations: TNF, tumour necrosis factor; TRADD, TNFRSF1A-associated via death domain; TRAF, TNF receptor-associated factors; cIAP, cellular inhibitor of apoptosis protein; CYLD, deubiquitinase cylindromatosis; FADD, FAS-associated death domain; MLKL, mediator mixed-lineage kinase domain like; RIPK, receptor-interacting protein kinase; PGAM5, phosphoglycerate mutase 5; Drp1, dynamin-related protein 1; ROS, reactive oxygen species; TLR3/4, TNF-like death receptors 3/4; PAMPs, pathogen-associated molecular patterns; LPS, lipopolysaccharide. Figure is modified from References [84,91,92].

The tumour necrosis factor alpha (TNFα)-mediated pathway of necroptosis has been most extensively investigated. The initial steps of cell death in TNFα-mediated necroptosis are shared by apoptosis and nuclear factor kappa light chain enhancer of activated B cells (NF-κB) signalling. Upon TNFα activation by death inducer, it binds to TNFR1 and forms a death receptor associated complex I at the plasma membrane [80] (Figure 2). This complex I binds to TNFR-associated death domain (TRADD) which then recruits receptor-interacting protein kinase 1 (RIPK1) through homophilic interaction between its death domain and binding to adaptor protein TNFR-associated factor 2 (TRAF2) [80]. Further, cellular inhibitor of apoptosis 1 (cIAP1) is recruited by RIPK1 to stabilise the complex I. This complex consists of TNFR1, TRADD, TRAF2, cIAP1 and RIPK1. Polyubiquitylation of RIPK1 in complex I recruits and activates transforming growth factor β (TGFβ)-activated kinase1 (TAK1), TAK1- binding proteins (TAB1 and TAB2) along with inhibitor of NF-κB kinase (IκK) which leads to the activation of NF-κB pathway [81]. Cylindromatosis (CLYD) results into deubiquitination of RIPK1, translocating into the cytoplasm to form another cytosolic death-inducing complex, termed complex II [82,83]. Complex II consists of RIPK1, TRADD, fas-associated protein with death domain (FADD) and CASPASE8. At this stage, CASPASE8 determines whether to initiate apoptosis and inhibit necroptosis [84]. However, if CASPASE8 is inhibited or inactivated by either chemical or viral inhibitors, RIPK1 will interact with RIPK3 forming amyloid fibrils downstream of TNFR1 at complex II [85]. The auto-interaction of RIPK3 will instigate recruitment and phosphorylation of MLKL [85,86]. This complex is known as the necrosome [85]. Finally, MLKL translocates to the plasma membrane and causes membrane permeabilization leading to necroptotic cell death [86,87,88]. Until recently, MLKL activation was considered to be the final stage of necroptosis; however, the rapid influx of Ca^2+^ into the cells after activated MLKL is followed by rapid exposure of phosphatidylserine before the loss of membrane integrity [89]. The fate of the cell at this stage depends on the function of endosomal sorting complexes required for transport (ESCRT) III [89]. Activation of MLKL results into formation of small bubbles at the plasma membrane which is ultimately controlled by ESCRT-III. ESCRT-III is required to maintain cell viability [90] and to delay the plasma membrane permeabilization and cell death following MLKL oligomerization [89]. Subsequently, this delay provides enough time for cells to release damage signals such as cytokines, chemokines as well as activation of CD8+ T cells before its demise [89].

#### 6.1.3. The Core Members of Necroptotic Machinery: The Executors

##### RIPK1/RIP1

Receptor-interacting protein kinase 1 (RIPK1/RIP1) along with its kinase activity plays a key role in cell death via RIPK3/MLKL-dependent necroptosis [93,94]. Receptor-interacting protein kinase 1 is a member of the receptor-interacting serine/threonine kinase family and consist of three domains: N-terminal kinase domain, intermediate domain and a carboxy-terminal death domain [95]. It is the presence of an RIP homotypic interact motif (RHIM) motif at the intermediate domain that mediates its interaction with downstream RIPK3 [96]. The C-terminal death domain can interact with death receptor protein at the cytoplasmic membrane and instigate apoptosis and regulate caspase-dependent apoptosis [97].

Evidence to support RIPK1 involvement in necroptosis comes from a study using Jurkat T cells with an RIPK1 mutant study [94]. Holler et al. howed that the presence of RIPK1 is inevitable in TNFα-induced necrotic death. Upon activation of cell death by FAS, TNFα or TRAIL, RIPK1 mutant Jurkat T cells fail to undergo caspase-independent necrotic death. Moreover, TLR-induced necroptosis increased protein levels of RIPK1 [82]. Interestingly, some proteins assist RIPK1–RIPK3 to execute necroptosis in a programmed manner. The RIPK1/3 kinase activation was facilitated by deubiquitylation of RIPK1 by CYLD and, hence, results in necroptosis [98,99]. In relation to liver injury, the RIPK1 protein level was upregulated in a bile duct ligation (BDL) model in mice [100]. Moreover, RIPK1 involvement has been suggested in the pathogenesis of NAFLD, as Majdi et al. found an increased level of RIPK1 in the serum samples of NAFLD patients [101].

##### RIPK3/RIP3

Receptor-interacting protein kinase 3 (RIPK3/RIP3) has emerged as a central player of necroptosis in the past few years. The location of the human *RIPK3* gene is on chromosome 14 and is known to encode a protein of 518 amino acids and its N terminal has an active kinase domain [102]. The C terminal of each RIP kinase is unique and is responsible for its participation in signalling cascades [96]. Other RIP kinases, such as RIPK1, contain a death domain, and RIPK2 contain a caspase recruitment domain (CARD). RIPK3 contains a unique degenerated C-terminal motif known as RHIM which is also present in RIPK1 intermediate domain. This RHIM mediates protein–protein interaction and necrosis [96]. The involvement of RIPK3 in necroptosis was initially observed by Feng et al. [103] in 2007. The role of RIPK3 and CASPASE8 inhibition in necroptosis has been demonstrated in *Caspase8* knockout mice models. Kaiser et al. have shown the lethality of *Caspase8* knockout embryos at the mid-gestational stage [104]. On the other hand, double knockout (*Caspase8^-/-^Ripk3^-/-^*) mice survived, indicating the involvement of necroptosis [104]. Further, studies have shown that necroptosis occurs independently from caspase activity and cannot be blocked by zVAD-fluoromethyl ketone (zVAD), a caspase inhibitor [64]. In another study, when CASPASE8 activity was blocked, RIPK1 and RIPK3 participated in protein–protein interaction through their RHIM motif. This led to individual phosphorylation of RIPK1, RIPK3 and MLKL and resulted in necroptosis [75,86].

There have been a growing number of studies indicating the involvement of RIPK3 in liver injury. For instance, RIPK3 medicated necroptosis has been associated with ethanol-induced hepatocyte injury [105]. Moreover, in a chronic alcohol feeding plus binge feeding murine model, significant upregulation of RIPK3 was noted, whereas RIPK3 knockout mice showed decreased liver alanine aminotransferase (ALT) levels [106]. In addition, a methionine- and choline-deficient (MCD)-diet induced hepatic necroptosis was attenuated in RIPK3 deficient mice [107]. Likewise, hepatocellular necrosis was significantly decreased in RIPK3 knockout mice subjected to BDL suggesting the involvement of necroptosis during BDL-induced injury [100]. Further, necroptosis is known to decrease liver function and result in organ failure [49,108]. FAS/RIPK1/RIPK3-mediated necroptosis was observed in acute liver injury induced by concanavalin A in mice [109]. It was found that CASPASE8 deletion in hepatocytes triggered the necrotic form of liver injury following concanavalin A induction and demonstrated FAS/RIPK1/RIPK3 complex activation [109]. 

Involvement of RIPK3 in other organ systems has been extensively studied. Cho et al. have shown that RIPK3 is a crucial activator of necroptosis induced by TNFα [63]. They demonstrated that phosphorylation of RIPK3 and RIPK1 triggered a pronecrotic kinase cascade activation. Similarly, He et al. also demonstrated that RIPK3 kinase activity is a key mediator of necroptosis [72]. RIPK3 knockdown in the human colon cancer cell line (HT-29) revealed the absence of cell necrosis and cell survival upon application of cell death inducer TNFα/second mitochondria-derived activator of caspase (SMAC)/zVAD compared to control HT-29 cells [72]. Similarly, in NIH-3T3 cells, RIPK3 is required to switch between TNFα-induced apoptosis and necrosis [110]. RIPK3 is also required in TNFα + zVAD-mediated necrosis. In a separate study, the initiation of virus-induced tissue necrosis and inflammation was attenuated in RIPK3 knockout mice [63]. Further, crystal-induced tissue injury has also been shown to induce RIPK3- and MLKL-dependent necroptosis in mouse kidney and human synovial fibroblasts [111].

#### 6.1.4. The Effector

##### MLKL

Mixed lineage kinase domain-like protein (MLKL) is a protein in the necroptotic signalling cascade, functioning downstream of RIPK3 [87,88]. The MLKL protein contains two domains: a N-terminal domain consisting of four helix bundles and a C-terminal pseudo-kinase domain [86,112]. This pseudo-kinase domain acts as a molecular switch that determines active or inactive MLKL configuration. Hence, its C-terminal interconnects with RIPK3 during necroptosis [87]. In contrast to RIPK3, which is functionally active, MLKL is related to a group of proteins that are enzymatically inactive, hence, termed pseudokinase [113,114]. When the phosphorylated RIPK3 propagates to form the necrosome, it recruits MLKL. Finally, upon activation, MLKL migrates to the plasma membrane which disintegrates releasing cellular contents and resulting in necroptotic cell death [115,116]. A study conducted by Wang et al. showed that necrotic cell death in drug-induced liver injury was due to the fact of MLKL activation [116]. In the context of inflammatory liver diseases, in both human samples and mouse models, MLKL activation is strongly correlated with hepatocellular necrosis [117]. Recent reports have also revealed a high expression of MLKL in human liver samples from patients with primary biliary cholangitis indicating activation of necroptosis. Similar results were seen in the murine model of BDL [100]. In other study, dermal fibroblast cells derived from *Mlkl^-/-^* mice, showed resistance to induced necroptosis [118]. The *Fadd^-/-^Mlkl^-/-^* double knockout mouse embryonic fibroblasts were resistant to TNFα plus zVAD-induced necroptosis [119]. The siRNA knockdown of MLKL in HT-29 cells ceased the progression of necrosis signalling [88].

## 7. Necrosis in Liver IR Injury

The mechanism of hepatic cell death after IR is controversial. Initial studies suggested that hepatocyte cell death is via necrosis with the release of liver enzymes during liver IR injury [120]. However, by the end of 20th century, as tools have developed to recognize apoptotic cell death, the views on the mechanism of cell death in liver IR injury quickly shifted toward apoptosis [121]. Studies have emerged demonstrating the involvement of apoptosis as shown by terminal deoxynucleotidyl transferase deoxyuridine triphosphate nick end labelling (TUNEL) staining and caspase activity [122,123,124] as well as a protective role of caspase inhibitors in hepatic IR injury [125,126]. In contrast, a comprehensive study performed by analysing the hallmark features of apoptosis, such as nuclear condensation, cell shrinkage and apoptotic bodies along with caspase-3 activity, showed only a small increase in the number of apoptotic cells after only one time point (1 h) of reperfusion and at other reperfusion time points, apoptotic cell number did not exceed baseline levels, whereas the counts of necrotic cells gradually increased even at 24 h of reperfusion [127]. This study reported only 0.3% of hepatocytes were deemed apoptotic verses 60% necrosis and, overall, >95% of damaged cells died via necrosis post IR injury. Further use of a caspase inhibitor in this study did not reduce the ALT level, DNA fragmentation or necrotic area. Interestingly, most necrotic hepatocytes were also positively stained in the TUNEL assay [127]. This finding raised further concerns that TUNEL staining, which is presumably used as marker for apoptosis, can also be detected in necrotic cell death as DNA degradation could also result after necrosis [128,129,130]. In addition, since apoptotic cells are visible for only a limited time in tissue sections compared to necrotic cells, analysis of plasma biomarkers of apoptosis and necrosis become useful to understand the underlying mechanism. Plasma biomarkers of necrotic cells (micro-RNA122, full-length cytokeratin 18 (FK18) and high mobility group box 1 (HMGB1)) showed significant elevation after IR with supportive histology when compared to the biomarkers of apoptosis (caspase-3 activity and caspase-cleaved fragment of cytokeratin 18 (CK18)) [131]. However, no morphological evidence of apoptotic cell death as well as absence of caspase-3 activity in plasma was reported after the same duration of IR injury. Quantitative comparative analysis of FK18 (necrosis marker) and CK18 (apoptosis marker) in the same study revealed necrosis is predominant during IR where apoptosis showed only a minor increase.

Many other studies in liver IR have demonstrated cell death via necroptosis without the activation of apoptotic markers [8,9,130,131]. Emerging studies have suggested that necroptosis plays an instrumental role and contributes to liver damage in steatotic livers following IR injury [8,9]. Koichi et al. revealed that rats with hepatic steatosis showed elevated levels of TNFα and necrotic areas in fatty liver tissue after inducing IR injury [132]. In another study, TNFα induced cell death in fat-loaded primary rat hepatocytes showed activation of necroptosis when CASPASE3 activity was inhibited [107]. Downregulation of nuclear HMGB1 using siHMGB1 in mouse IR injury resulted in protection from liver injury and decreased inflammation after liver IR [133]. Significant upregulation of both RIPK1 and RIPK3 has been reported after IR injury with elevated level of TNFα and Interlukin-6 (IL-6) [134]. Whereas some studies demonstrated contradicting results that necroptosis is not active in IR injury using similar in vivo models [135,136]. This conflict could possibly be the result of variation in experimental models and requires further analysis.

Studies have demonstrated the critical involvement of RIPK3 in IR injury in various organ systems apart from liver. RIPK3 overexpression was seen in an in vitro model of hippocampal neuronal HT-22 cells undergoing IR injury [137]. In the same study, an in vivo experiment also showed the RIPK3 and MLKL overexpression in the middle cerebral artery occlusion (MCAO) mouse model of ischaemia brain injury. Similarly, RIPK1/RIPK3/MLKL-mediated necroptosis played a critical role in rat nucleus pulposus (NP) cell death induced by compression, a mechanical stress [138].

## 8. Necroptosis in Steatotic Hepatic Injury and Steatotic IR Injury

Based on histological scoring, NAFLD is defined as the presence of ≥5% hepatic steatosis in the liver [139]. Some studies showed the absence of RIPK3 protected against liver injury, steatosis, inflammation, fibrosis and oxidative stress in an MCD-diet model [140]. However, necroptosis has been linked to both NAFLD and alcoholic liver diseases [105,107,141]. The initial study of necroptosis in NAFLD showed the upregulation of RIPK3 in NASH patients and in a mouse model using the MCD diet that contributed to liver injury and liver fibrosis [141]. The RIPK3-dependent necroptosis resulted in liver injury, inflammation and liver fibrosis. Similarly, increased RIPK3 and MLKL levels were observed in NAFLD patients and in high-fat choline-deficient (HFCD) mouse models [107]. These studies have shown the profound involvement of necroptosis in steatotic liver injury.

Studies in murine models have suggested that hepatocyte injury and inflammation are the primary contributors that aggravate the injury in steatotic liver IR injury [142,143,144]. Li et al. have shown an increase in necrotic area along with increases in TNFα, IL-6 and interferon-γ (IFN-γ) after IR injury in methionine, choline-deficient and high-fat (MCDHF) diet fed mice [143]. Another study also reported increased levels of TNFα and Interlukin-1β (IL-1β) mRNA in obese mice compared to lean controls [142]. Necroptosis in steatotic liver IR has been recently studied using rat and mouse models where hepatic ischaemia was induced by occluding vessels supplying the median and left lateral lobe for various periods and liver injury was monitored following different reperfusion periods [8,9]. Two different studies using different animal models have shown the upregulation of RIPK3 and MLKL resulting in necroptotic cell death of hepatocytes following IR injury. Kim et al. demonstrated that mice fed a high-fat diet (HFD) demonstrated increased RIPK3 and MLKL expression after IR compared to chow diet in a rat model [8]. These findings were later supported by Sun et al., who found both RIPK3 and MLKL were significantly upregulated after IR injury in western diet (WD)-fed mice compared to the chow diet. Exacerbation of injury in WD mice was also noted which was significantly reduced in Mlkl^-/-^ mice [8,9]. However, these studies have not used the moderately steatotic liver which contributes to the large pool of discarded livers in the human setting and could potentially become a likely candidate for transplantation given the current donor shortage. Furthermore, investigation of IR injury in combination with a macrovesicular steatotic model also becomes essential, as studies have shown that such livers are more susceptible to IR injury compared with microvesicular steatosis. Interestingly, ferroptosis, an iron-dependent non-apoptotic cell death pathway, has been linked to hepatic IR injury [145]. A recent study in a murine model of hepatic IR injury has shown the upregulation of the ferroptosis marker prostaglandin-endoperoxide synthase 2 (*Ptgs2)* after IR insult which was prevented by the ferroptosis inhibitor, ferrostatin-1 (Fer-1) or α-tocopherol [145]. More studies are indeed required to investigate the involvement of ferroptosis during steatotic liver undergoing IR injury. Further, with the emergence of studies uncovering co-regulation and cross-talk among various cell death pathways, such as pyroptosis, apoptosis, and necroptosis, which is termed PANoptosis [146,147], more studies need to be conducted to investigate the possible co-existence of multiple cell death pathways during hepatic IR injury.

## 9. Immune Response and Inflammation During Necroptosis

During oxidative stress or in ischaemic conditions, tissue damage or dying cells can release endogenous molecules DAMPs as warning signals (alarmins) for immune responses [148,149,150]. The elevation of these signals in response to ischaemia is clinically relevant, as they may indicate tissue at risk of further injury. During IR injury, ROS and ER stress can work together to facilitate the release of DAMPs [151,152]. Some studies have suggested the release of DAMPs during necrosis or secondary necrosis (Table 1). Since release of DAMPs during apoptosis is absent or minimal, their secretion is typically considered a major necroptotic event [77]. The necroptotic mediator, RIPK3, is known to facilitate the traffic of DAMPs to the extracellular space after MLKL activation [77]. Many studies in liver and other organs have provided evidence of release of DAMPs in IR injury and various well-defined DAMPs, such as HMGB1, interleukin-33 (IL-33), ST2, interleukin-1α (IL-1α) and ATP, are known to be involved during necroptotic IR injury. Some of these are discussed below.

### 9.1. HMGB1

High mobility group box 1 (HMGB1) is a highly conserved nuclear protein found in all multicellular organisms [153,154]. As it consists of DNA-binding domains, it orchestrates DNA binding as well as stabilization of nucleosomes to aid in transcriptional regulation of genes [155,156]. Importantly, HMGB1 can also function as an extracellular inflammatory mediator and hence it is also considered an alarmin [157]. Untimed cellular death caused by stress or trauma can result in passive release of HMGB1 through the cytoplasm into the extracellular space alerting the immune system [153,154]. Interestingly, during apoptotic cell death HMGB1 remains tightly bound to chromatin and, hence, is not released from the nucleus [158]. Since cells release HMGB1 during necrotic cell death rather than during apoptosis, detection of HMGB1 in the extracellular environment can be interpreted as a distinct feature of necroptosis. Further, early active release of acetylated HMGB1 by hepatocytes undergoing IR insult has been described previously (Table 1) [154]. Further HMGB1 expression was increased in steatotic liver compared to lean liver in the study conducted in rat model [132].

### 9.2. Interlukin-33 

Interlukin-33 (IL-33) is a nuclear protein and is a member of the cytokine IL-1 family [159]. As in the case of HMGB1, IL-33 also has a dual function: as a chromatin-associated protein to participate in gene repression [155] and as a proinflammatory mediator [155,159]. IL-33 released to the extracellular environment, mediates its activity via IL-1 receptor like 1 ST2 protein [155]. During mechanical trauma, IL-33 is systemically released as an alarmin signal [160]. IL-33 activity is inhibited by caspase activation and, hence, IL-33 expression is inactivated during apoptotic cell death [159]. Release of endogenous IL-33 is known to contribute during kidney IR insult [161] and absence of IL-33 or ST2 protected livers from IR injury [162] (Table 1).

### 9.3. Proinflammatory Cytokines

IR injury in solid organs contributes to the rapid release of proinflammatory cytokines including TNFα, IFNγ, IL-1β, IL-6 and interleukin- 8 (IL-8). For instance, in a study conducted in a murine model of IR renal injury, significant upregulation in IL-6, interleukin- 11 (IL-11), leukaemia inhibitor factor (LIF) and macrophage inflammatory protein (MIP-2) was observed [163]. In a liver transplantation study conducted in pigs, increased levels of TNFα and IL-6 were seen after IR injury (Table 1) [164]. The increased level of TNFα was noticed in hepatic IR injury in a mouse model [165]. Further, systematic upregulation of IL-1 and TNFα were seen during renal IR injury in mouse models [166]. Ischaemia-induced injury in rat kidney showed increased levels of cytokines such as IL-2, IL-10, IFN-γ and transforming growth factor β (TGF-β) [167]. IL-1β, Il-1, Il-6 and TNFα levels were found to be comparatively higher in liver preservation solution effluent than in donor and recipient prior to the surgery [168]. In another study, IL-8 levels were significantly upregulated in cardiac transplantation patients [169]. In the same study, human saphenous vein endothelial cell culture in hypoxic conditions increased levels of IL-8 in a time-dependent manner.

In summary, the detection of circulatory levels of HMGB1, IL-33 and proinflammatory cytokines could potentially be used as effective tools to study the role of alarmins during necroptosis in liver IR injury. Moreover, levels of alarmins could play key role in predicting injury risk and also be a potential therapeutic target.

## 10. Necroptosis Inhibitors

The discovery of inhibitory molecules that are able to attenuate necroptotic cell death has translational potential. Hence, investigation of necroptotic inhibitors is essential. Several necroptosis inhibitors have been recently reviewed elsewhere (Table 2), and here we review some of most thoroughly investigated inhibitors of necroptosis in more detail.

### 10.1. RIPA-56

RIPA-56 is a metabolically stable and highly potent RIPK1 inhibitor, targeting the activity of MLKL [208]. In a study conducted in a NASH mouse model to investigate the effect of RIPA-56 in both prophylactic and curative setting, RIPA-56 treatment attenuated the liver injury, inflammation, hepatic steatosis and fibrosis [101]. In the same study, while a HFD diet upregulated the expression of RIPK3 and MLKL and increased ALT level, treatment with RIPA-56 at prophylactic and curative levels suppressed their expression. Further, RIPA-56 decreased the intracellular fat accumulation in an in vitro model of hepatocytes [101]. As discussed earlier, steatotic liver is remarkably vulnerable to IR injury, and since RIPA-56 is effective in depleting intracellular lipid droplets, it is therefore important to further investigate the use of RIPA-56 in steatotic livers during IR.

### 10.2. Necrostatin-1

Necrostatin-1 (Nec-1), a RIPK1 kinase inhibitor, has been effectively used as a potential therapeutic, targeting RIPK1 activity in necroptosis [63,72]. Necrostatin-1 is a potent small molecule originally identified during screening of a chemical library [74]. Necrostatin-1 leaves RIPK1 in an inactive state by preventing the phosphorylation as well as ubiquitination during necrosome formation [209]. Evidence supporting the therapeutic use of Nec-1 has been studied in various in vitro and in vivo models. IR-induced liver injury and ROS production was reduced after pre-treatment with Nec-1 in a HFD-fed mouse model [210]. Further, TNFα-induced injury was minimised by Nec-1 treatment in an in vitro study using primary mouse hepatocytes [210]. Necrostatin-1 has also been shown to decrease hepatic triglyceride level in an obese mouse model [211]. Necroptosis in retinal ganglion cell 5 (RGC-5) cells followed by oxygen glucose deprivation (OGD) has been significantly reduced by pre-treatment of cells by Nec-1 [212]. Further, Nec-1 is shown to inhibit ischaemic brain injury in mice [74]. Similarly, Wang et al. demonstrated that Nec-1 reduced RIPK3 expression in ouabain-induced injury in primary culture of rat cortical neurons [116]. Moreover, RIPK1–RIPK3 interaction was also found to be inhibited by Nec-1 [63]. However, Nec-1 can inhibit indoleamine 2,3-dioxygenase, an enzyme known to participate in both innate and adaptive immunity [213]. Furthermore, it can also function as a ferrostatin [214] and research has shown its lethal effect in in vivo studies [215]. Hence, further investigation is required to understand its effect during IR injury in steatotic liver.

### 10.3. GSK’872

GSK’872 is described as an RIPK3-selective kinase inhibitor and has been effectively used as a potential therapeutic target of RIPK3 activity during necroptosis. Pre-treatment with GSK’872 decreased the IR liver injury as well as ROS level in an HFD-fed mouse model [210]. In a study performed in J774 murine macrophages, GSK’872 treatment blocked TRIF-induced necrosis [216]. The same authors claimed that TNFα-induced cell death in 3T3-Sa cells was remarkably reduced in a dose-dependent fashion after GSK’872 administration. In a separate study, an in vitro model using HT-22 cells and an in vivo study in a mouse model, GSK’872 treatment suppressed ischaemic brain injury induced necroptotic cell death by alleviating RIPK3 expression and improved cell viability [137]. Interferon-β (IFN-β) activated-cell death in RIPK1 knockout mouse embryonic fibroblasts was also suppressed by the administration of GSK’872 [217].

### 10.4. MLKL Inhibitors

The human specific MLKL inhibitor, necrosulfonamide (NSA) is known to halt necroptosis in in vitro models [87]. A study has also shown treatment with NSA significantly reduced the lipid droplets in fat-induced primary human hepatocytes [218]. Because the steatotic liver possesses an increased risk during IR, the use of NSA in steatotic liver undergoing IR injury could potentially become an important investigation. Another MLKL inhibitor, compound 1 (also referred to as GW806742X or SYN1215) has shown to prevent necroptosis in an in vitro study performed using mouse fibroblasts by targeting pseudokinase domain of MLKL [112]. However, NSA is human-specific and further investigation has not been performed in murine models [87]. Similarly, the mechanism of action of compound 1 is unclear as its protective feature involves both interaction with MLKL as well as inhibition of RIPK1 [112].

Though RIPA-56 and Nec-1 have been investigated in NASH and NAFLD models, more research is needed to evaluate their potential effect during steatosis as well as during IR injury. Apart from the abovementioned inhibitors, some potential drugs to block necroptosis have been clinically used with US Food and Drug Administration approval, namely, dabrafenib, a selective RIPK3 inhibitor that alleviates acetaminophen (APAP)-induced liver injury; sorafenib, which can inhibit the kinase activity of both RIPK1 and RIPK3; pazopanib, a RIPK1 inhibitor; and ponatinib, a RIPK1 and RIPK3 inhibitor. These are some drugs that are in use for other liver indications [207]. However, none of these drugs have been used for direct treatment of necroptosis-related illness or liver IR injury in the clinic. More studies are necessary to evaluate the potential therapeutic efficacy of necroptotic inhibitors in the liver transplantation setting.

## 11. Organ Preservation Strategies and Defatting of Steatotic Liver during Liver Transplantation

During transplantation, donor liver preservation is a critical step to maintaining the quality of liver as well as to improve post-transplant outcomes. Conventional static cold storage (SCS) is considered to be the primary method for organ preservation [219,220]. However, if it is prolonged, this method does not provide sufficient protection against IR injury particularly for the marginal livers such as steatotic livers and DCD livers which are already in a vulnerable state. As a result, alternative strategies, such as machine perfusion (MP), have been proposed to reduce the harmful effect of SCS and to better assess the organ quality [221,222,223]. Machine perfusion methods mimic the physiological process by establishing controlled continuous flow of nutrients and antioxidants and promoting the flushing of inflammatory cytokines and toxins from the graft [224]. Hence machine perfusion is considered superior to SCS.

Studies have been conducted to investigate the potential use of MP in steatotic liver grafts [224,225]. In a study conducted in macrosteatosis murine model, the use of MP restored graft viability compared to SCS [225]. Another study has revealed the protective nature of MP in marginal livers by reducing cytokine-mediated IR injury [224]. Hypothermic machine perfusion (HMP) is one of the suggested methods for graft preservation where organs are maintained at lower temperature to reduce cellular metabolism. In human clinical studies, HMP significantly reduced proinflammatory cytokines and oxidation markers [226]. Likewise, use of HMP in pig DCD liver grafts showed reduction of necrosis as well as improved bile flow and ATP level [227]. However, prolonged organ storage with cold perfusion could potentially damage the sinusoidal endothelium and endoplasmic reticulum [228]. Another preservation technique, venous systemic oxygen persufflation (VSOP) was developed by Minor et al. [229] which demonstrated that VSOP can reduce liver necrosis as well as improve cellular autophagy in steatotic livers and also maintain the sinusoidal endothelial lining during IR exposure [230,231]. Ischaemic preconditioning (IPC) is another protective strategy where a short ischaemic phase is followed by a brief reperfusion interval, before storing the graft in a long ischaemic state. IPC has shown to reduce preservation injury in steatotic livers, decrease parenchymal necrosis and reduced postoperative enzyme release [232,233]. However, contradicting studies also showed higher enzyme release in recipients post-transplantation [234].

The arrival of normothermic machine perfusion (NMP) that maintains the liver grafts at physiological temperatures to support homeostasis has become a promising preservation technique. NMP aims to minimise the IR injury in liver graft and improve preservation of ECD livers [235]. The successful use of NMP in liver transplantation of ECD livers provides the opportunity for therapeutic intervention prior to transplantation of ECD livers [236]. NMP not only easily facilitates addition of therapeutic agents, such as necroptosis inhibitors described above, to the perfusion system but allows assessment of hepatocellular function and injury prior to transplantation. In addition, monitoring of DAMP/alarmin release is feasible during NMP and may allow development of predictive biomarkers to identify ECD livers that could be successfully transplanted. Studies have been conducted to examine the utilisation of NMP in steatotic liver grafts [237,238,239,240]. A steatotic porcine model study has revealed that under NMP, the steatotic liver was capable of maintaining perfusate base excess, factor V and bile production as well as decreasing macrosteatosis [237]. Interestingly, another study investigating the use of a cocktail of defatting agents during NMP in Zucker rats showed intracellular lipid content of perfused liver was decreased by 50% and increased lipid oxidation [238]. Likewise, when L-carnitine was added to a cocktail of defatting agents a reduction of macrosteatosis from 42.5% to 8.5% after NMP was observed [241].

Though animal studies have shown positive outcomes of the use of NMP alone or in combination with defatting agents, similar results have not been seen in the studies using human steatotic liver involving NMP alone, as it did not decrease the percentage of macrosteatosis when compared with SCS [242]. These data indicate that there is a necessity of combination of defatting agents along with NMP. Boteon et al. had successfully showed increased hepatic lipid metabolism and a reduction in macrosteatosis after NMP with defatting molecules in a study conducted in human discarded livers [239]. Aoudjehane et al. also showed treatment with NSA, a MLKL inhibitor, in combination with or without a defatting agent reduced the intracellular lipid droplet in an in vitro steatosis model [218]. With increasing evidence of involvement of necroptosis in steatotic liver IR, it will be worth investigating the combination treatment of necroptosis inhibitors along with NMP in steatotic livers during IR exposure.

In summary, the abovementioned studies of different organ preservation methods have provided a platform to study the potential use of organ preservation strategies for steatotic donor liver during transplantation.

## 12. Conclusions

Many interesting studies expanding our view on necrosis and necroptosis have been described in the past decade. Necroptosis, a novel regulated form of cell death, has been linked to IR insult where RIPK3 and MLKL are known to play fundamental roles. Liver transplantation has been regarded as an only treatment option for patients with end-stage illness and the shortage of donor livers has emphasised the use of extended criteria donor livers, such as steatotic livers to increase liver transplantation rates. Unfortunately, steatotic livers are associated with primary graft non-function, early allograft dysfunction and graft loss, as they are susceptible to IR injury. Hence, it is important to further investigate the role of necroptosis during IR especially in moderately steatotic livers with macrovesicular steatosis which seems to confer more susceptibility when compared to microvesicular steatosis. Moreover, identification of alarmins as potential biomarkers to predict the outcome of transplantation may become even more important as there are limited therapeutic options. Further research is needed to accelerate the understanding of the mechanisms and development of therapeutic agents and organ preservation strategies to reduce IR injury in steatotic liver, all with the overall goal being to expand the pool of donor organs available for liver transplantation.

## Figures and Tables

**Table 1:** Overview of DAMPS associated with different types of cell death. The table is modified from Reference [170].

DAMPs	Type of Cell Death Involved	Involved in IR	References
ATP	Apoptosis, necroptosis, accidental necrosis and immunogenic apoptosis (either pre-apoptotic or early apoptotic active secretion)	Yes	[170,171,172,173,174,175]
Cyclophilin A	Necrosis, necroptosis	Not defined	[170,176,177]
F-actin	Necroptosis, accidental necrosis and secondary necrosis (exposure following cell membrane permeabilization)	Not defined	[170,178,179]
HSP70, HSP60, HSP72, GRP78 and GP96	Necroptosis, necrosis (passively released) and immunogenic apoptosis (either pre-apoptotic or early or mid-apoptotic surface exposure)	Yes	[66,170,174,175,180,181,182]
Histones	Accidental necrosis, apoptosis	Yes	[170,183,184,185]
HMGB1	Necroptosis, accidental necrosis and immunogenic apoptosis (secondary necrosis, passively released), cell death accompanied by autophagy	Yes	[153,155,170,175,180,186]
HMGN1	Necroptosis, secondary necrosis (passively released)?	Not defined	[66,170,187,188]
IL-1α	Necroptosis, accidental necrosis (passively released)	Yes	[170,189,190]
IL-33	Necroptosis, accidental necrosis (passively released)	Yes	[170,186,191]
IL-6	Necroptosis	Yes	[66,164,183,192]
Mitochondrial DNA	Accidental necrosis (passively released)	Yes	[70,170,193]
Mitochondrial transcription factor A	Accidental necrosis (passively released)	Yes	[170,192,194]
Monosodium urate	Accidental necrosis (passively released)	Not defined	[170,195]
Reactive carbonyls and oxidation-specific epitopes	Apoptosis or necrosis induced by ROS-producing agents	Not defined	[170,196,197,198]
Ribonucleoproteins, mRNA and genomic DNA	Accidental and secondary necrosis (passively released)	Not defined	[170,175,199,200,201]
S100A8, S100A9 and S100A12	Accidental necrosis (passively released)	Yes	[170,175,202,203,204]
HSP90	Apoptosis, necroptosis	Yes	[66,170,201,205,206]
IL-1b	Necroptosis, apoptosis	Yes	[66,170,183,189]

Abbreviations: DAMPs, damage-associated molecular patterns; ATP, Adenosine triphosphate; HMGB1, high mobility group protein B1; HMGN1, high mobility group nucleosome binding domain 1; GRP, glucose regulated protein; HSP, heat shock protein; IL, interleukin; ROS, reactive oxygen species.

**Table 2:** List of necroptosis inhibitors. The table is modified from Reference [207].

Intervention/ Inhibitors	Target
Nec-1s	RIPK1
GSK872	RIPK3
GSK’843	RIPK3
Dabrafenib	RIPK3
Necrostatins (1/3/4/5)	RIPK1
Tozasertib	RIPK1
Sunitinib	RIPK1
GSK3145095	RIPK1
GSK’963	RIPK1
GSK’547	RIPK1
RIPA-56	RIPK1
Sibiriline	RIPK1
Compound 4b	RIPK1
Necrosulfonamide	MLKL
Compound 1	MLKL
Ponatinib	RIPK3
Sorafenib	RIPK3

## Abbreviations

IR	ischaemia-reperfusion
RIPK3	receptor-interacting protein kinase 3
MLKL	mixed lineage kinase domain-like pseudokinase
NAFLD	non-alcoholic fatty liver disease
ECD	extended criteria donors
PNF	primary non-function
DCD	donation after cardiac death
ATP	adenosine triphosphate
ADP	adenosine diphosphate
AMP	adenosine monophosphate
Na^+^- K^+^	sodium potassium
Ca^2+^	Calcium
ER	endoplasmic reticulum
H^+^	hydrogen ion
ROS	reactive oxygen species
MnSOD	superoxide dismutase
GPx	glutathione peroxide
OH^.^	hydroxy radical
DAMPs	damage-associated molecular pattern molecules
BDL	bile duct ligation
TNFR1	tumour necrosis factor receptor superfamily member 1
FAS	fas cell surface death receptor
ZBP1	Z-DNA binding protein 1
PRR	pathogen recognition receptor
TLR	toll-like receptor
TNFα	tumour necrosis factor alpha
TRADD	TNFR-associated death domain
RIPK1	receptor-interacting protein kinase 1
TRAF2	TNFR-associated factor 2
NF- κB	nuclear factor kappa light chain enhancer of activated B cells
cIAP1	cellular inhibitor of apoptosis 1
TAK1	transforming growth factor β (TGFβ)-activated kinase1
TAB	TAK1- binding proteins
IKK	NF- κB kinase
CLYD	Cylindromatosis
FADD	fas-associated protein with death domain
ESCRT	endosomal sorting complexes required for transport
TRIF	toll–IL-1 receptor domain-containing adaptor inducing IFN-β
PGAM5	phosphoglycerate mutase 5
Drp1	dynamin-related protein 1
NP	*nucleus pulposus*
CARD	caspase recruitment domain
RHIM	RIP homotypic interact motif
zVAD	zVAD-fluoromethyl ketone
HT-29	human colon cancer cell line
SMAC	second mitochondria-derived activator of caspase
TUNEL	terminal deoxynucleotidyl transferase deoxyuridine triphosphate nick end labelling
ALT	alanine aminotransferase
FK18	full-length cytokeratin 18
HMGB1	*high mobility group box 1*
GRP	glucose regulated protein
HSP	heat shock protein
HMGN1	high mobility group nucleosome binding domain 1
CK18	cytokeratin 18
MCAO	middle cerebral artery occlusion
MCD	methionine and choline-deficient
HFCD	high-fat choline-deficient
IL	interlukin
IFN-γ	interferon-γ
MCDHF	methionine, choline-deficient and high fat
HFD	high fat diet
WD	western diet
Ptgs2	prostaglandin-endoperoxide synthase 2
Fer-1	ferrostatin-1
LIF	leukaemia inhibitor factor
MIP-2	macrophage inflammatory protein
TGF-β	*transforming growth factor* β
Nec-1	necrostatin-1
RGC-5	retinal ganglion cell 5
OGD	oxygen glucose deprivation
NSA	necrosulfonamide
APAP	acetaminophen
SCS	static cold storage
MP	machine perfusion
VSOP	venous systemic oxygen persufflation
IPC	ischaemic preconditioning
NMP	normothermic machine perfusion
